# ^99m^Tc-Besilesomab-SPECT/CT in Infectious Endocarditis: Upgrade of a Forgotten Method?

**DOI:** 10.3389/fmed.2019.00040

**Published:** 2019-02-27

**Authors:** Caroline Bouter, Birgit Meller, Carsten O. Sahlmann, Johannes Meller

**Affiliations:** Department of Nuclear Medicine, Georg-August-University Göttingen, Göttingen, Germany

**Keywords:** SPECT/CT, infection, besilesomab, endocarditis, BW250/183

## Abstract

Infective endocarditis displays a serious condition with high mortality rates. Establishing a reliable diagnosis can be challenging. This study evaluates granulocyte imaging with ^99m^Tc-Besilesomab-SPECT/CT in order to determine the clinical value of the method and its possible redefinition through the addition of hybrid imaging. The study comprises 26 consecutive patients with suspected infectious endocarditis or prosthetic valve infection that underwent ^99m^Tc-Besilesomab-SPECT/CT in our facility between December 2016 and September 2018. ^99m^Tc-Besilesomab-SPECT/CT images were reviewed by two independent and blinded observers and results were evaluated by transesophageal echocardiography (TEE) and blood culture results as well as by pathological, bacteriological, and clinical findings. Target-to-Background-Ratios were calculated for semi-quantitative analysis. 13/26 patients were in a post-surgical stage. ^99m^Tc-Besilesomab-SPECT/CT was positive in 6 cases. All 6 cases were true positive confirmed by pathological or clinical findings according to the modified Duke Criteria for infective endocarditis. Remaining 19/26 cases were true negative. Target-to-Background ratios were significantly higher in patients that were visually scored positive compared to negative cases. Inter-observer agreement was very good of deciding whether a scan was positive or negative. Sensitivity of ^99m^Tc-Besilesomab-SPECT/CT was 86–100% and specificity was 100%. ^99m^Tc-Besilesomab-SPECT/CT is a useful imaging method for the diagnosis of endocarditis, especially in difficult cases with prosthetic valves or cardiac devices and inconclusive findings in echocardiography. The added value of SPECT/CT was mainly finding and localizing increased uptake at a certain valve, prosthesis, or device cable.

## Introduction

Infective endocarditis and prosthetic valve infections display serious conditions with high mortality rates. In patients with valve replacements endocarditis occurs in about 1–6% of cases with increasing incidence over the past decades (1).

Diagnosis of infectious endocarditis usually relies on clinical features supported by echocardiography or blood cultures. The Duke Criteria system helps establishing a final diagnosis showing a sensitivity of about 80%. However, due to inconclusive findings in echocardiography or blood cultures up to 24% of cases with suspected endocarditis remain misclassified using the Duke Criteria (2).

To date, the American Heart Association and the European Society of Cardiology recommends the use of the “modified Duke Criteria” in which diagnosis of endocarditis is “definite” if histology or cultures of intra-cardiac lesions are positive or if different combinations of major and minor criteria are present (1, 3, 4).

The 2015 guidelines of the European Society of Cardiology for the management of infective endocarditis proposed including imaging modalities to the diagnostic workflow. Gated cardiac computed tomography (cardiac CT) and, in cases of suspected prosthetic valve infections, radionuclide imaging with ^18^F-Fluorodeoxyglucose-Positron-Emission Tomography (^18^F-FDG-PET) and leucocyte SPECT were suggested. Cardiac CT can be used in addition to TEE showing complementary anatomical features or complications as systemic embolization (5, 6). However, this method can evaluate larger vegetations and paravalvular extensions of abscesses while it is not suitable to detect small vegetations.

Radionuclide imaging can be applied to detect neutrophilic granulocytic infiltration by *ex vivo* radiolabeled leucocytes and anti-leucocyte antibodies. The murine monoclonal IgG antibody BW250/183 (Besilesomab) detects granulocytes *in vivo* binding to CD66/67 (non-specific cross-reacting antigen 95). CD66/67 is expressed on mature granulocytes, and to a lesser extent on myelocytes and promyelocytes (7). ^99m^Tc-Uptake of Besilesomab is mainly triggered by binding to post-migrational granulocytes at the site of infection. However, data about the role of ^99m^Tc-Besilesomab-SPECT in the diagnosis of endocarditis remains scarce.

This study evaluates ^99m^Tc-Besilesomab-SPECT/CT in order to determine the clinical value of the method and its possible redefinition through the addition of hybrid imaging.

## Patients and Methods

### Patients

A total of 26 consecutive patients with suspected endocarditis that underwent ^99m^Tc-Besilesomab-SPECT/CT in our facility between December 2016 and September 2018 were evaluated retrospectively. The study comprised 8 women and 18 men aged 24–89 years (mean 69). 13/26 patients underwent cardiac surgery (valve replacement surgery or transcatheter aortic valve implantation (TAVI). Time between an intervention and SPECT/CT was 1–199 months (mean 49.5 months; Table 1).

**Table 1:** Patient characteristics.

	**SPECT/CT**
**Patient Characteristics**	
Patients (m/w)	26 (18/8)
Age (years)	24–89 (mean 69)
Cardiac surgery	13
Aortic valve replacement	10
Pulmonary valve replacement	1
Mitral/ tricuspid valve reconstruction	1
Cardiac device	1
Time between surgery and SPECT/CT (mean months)	49.5

All procedures involving human participants were in accordance with the ethical standards of the institutional and/or national research committee and with the 1964 Helsinki declaration and its later amendments or comparable ethical standards. The institutional review board approved this retrospective study. All patients signed an informed consent.

### Image Acquisition

In all patients a human anti-mouse antibody (HAMA) scan was performed prior to the examination. ^99m^Tc-Besilesomab-SPECT/CT was performed 4 and 24 h after injection of 653–807 MBq (mean 742 MBq) of ^99m^Tc-Besilesomab (Scintimun®, Iba/CIS Bio GmbH, Berlin, Germany). Planar images of the thorax, whole body images and SPECT/CT images were obtained at both time points. All patients were scanned on a GE Optima 640 SPECT/CT (GE Healthcare, Chicago, IL, USA). CT scans of the thorax were acquired with 120 kV and 2.5 mA, rotation time 1 s, pitch 1.25, and slice thickness 2.5 mm. SPECT images of the same field of view were acquired using a 128 × 128 matrix at the 140 keV photo peak with 6° per angle step and acquisition time of 30 s per angle step. Images were reconstructed using an ordered subset expectation maximization (OSEM) algorithm with 5 iterations and 10 subsets and a Butterworth filter.

### Interpretation of Findings

SPECT/CT images were analyzed using the Xeleris workstation with the Volumetrix software (GE Healthcare, Chicago, IL, USA). SPECT images were evaluated in coronal, transversal and sagittal planes as well as in maximum-intensity projection. In SPECT/CT scans co-registration of SPECT and CT images was verified and hybrid images were generated. Images were visually evaluated by two experienced nuclear medicine physicians. Observers were blinded to the medical history and results of other tests. Presence and location of focal abnormal uptake within the valve level, around valve replacements or around other cardiac devices were evaluated. Images were classified positive when at least one focus of abnormal uptake with an increase of contrast between images acquired 4 h p.i. and delayed images at 24 h p.i. was visible. Scans were classified negative when no sites of abnormal uptake were observed or uptake that was very low in contrast to the surrounding tissues (just above background level) with no time-dependent increase. Both observers scored the images from “0” to “3” as follows: 0 = no uptake; 1 = uptake just above background level with no time-dependent increase; 2 = distinct uptake above background level with high contrast to surrounding tissue and time dependent increase; 3 = intense uptake above background level with highest contrast to surrounding tissue and time dependent increase. Mediastinal uptake was used as background reference. Foci of abnormal uptake were matched to the anatomic localization using the CT images and image fusions.

Results of ^99m^Tc-Besilesomab-SPECT/CT were compared to pathological or bacteriological findings, TEE, blood culture results and clinical findings. Final diagnosis of endocarditis was defined according to the modified Duke Criteria as “definite,” “possible,” or “rejected” endocarditis. Final diagnosis was used in order to evaluate the ability of ^99m^Tc-Besilesomab-SPECT/CT confirming or excluding infectious endocarditis.

Target-Background-Ratios (TBR; Mean counts in the target/mean counts of the background) were calculated for semi-quantitative analysis. A target region of interest (ROI) was drawn around foci of abnormal uptake and mean counts were measured. In cases with no visible foci of abnormal uptake within the cardiac region a rectangle ROI was drawn around the suspected valve in respect to the clinical question and mean counts within this ROI were measured. In all cases a rectangle background ROI of 15 cm^2^ was drawn within the mediastinum and mean counts were measured.

### Statistical Analysis

All data are given as means ± standard error. Unpaired *t*-test, linear regression and Receiver Operating Characteristic (ROC) curve analysis was used for statistical evaluation. GraphPad Prism version 6.07 for Windows (GraphPad Software, San Diego, California, USA) was used for all calculations. In order to study intra-observer variability weighted Kappa was calculated using GraphPad QuickCalcs (GraphPad Software, San Diego, California, USA; https://www.graphpad.com/quickcalcs/kappa2/).

## Results

^99m^Tc-Besilesomab-SPECT/CT was performed in *n* = 26 patients (8 women, 18 men aged 24–89 years; mean age 69 years). *N* = 13 patients were in a post-surgical stage (aortic valve replacement *n* = 10; combined mitral and tricuspid valve replacement *n* = 1; pacemaker implantation *n* = 1; pulmonary valve implantation *n* = 1; Table 1) and 13 patients did not have any cardiac surgery prior to ^99m^Tc-Besilesomab-SPECT/CT. Transesophageal echocardiography (TEE) was available in 25/26 patients.

According to the modified Duke Criteria diagnosis was classified “definitive endocarditis” in 5 patients, “possible endocarditis” in 5 patients, and “rejected endocarditis” in 16 patients (Table 2).

**Table 2:** Modified duke classification.

	**Total**	**Positive ^**99m**^Tc-Besilesomab-SPECT/CT**	**Negative ^**99m**^Tc-Besilesomab-SPECT/CT**
**MAJOR CRITERIA**			
Positive blood cultures	10	6	5
**Staphylococcus aureus**	5	2	3
*Staphylococcus epidermidis*	1	1	–
*Enterococcus faecalis*	4	2	2
**IMAGING**			
Positive TEE	8	5	3
Vegetation	4	1	3
Abscess	2	2	–
Paravalvular thickening	2	2	–
Cardio CT	1	1	–
Paravalvular lesion	1	1	–
**PATHOLOGICAL CRITERIA**			
Histological proof of active endocarditis	2	2	–
**MINOR CRITERIA**			
Predisposing heart condition	13	5	8
Fever >38°C		2	2
Immunological phenomena	1	1	–
Osler's nodes	1	1	–
**MODIFIED DUKE CLASSIFICATION**			
Definitive endocarditis		5	–
Possible endocarditis		2	3
Rejected endocarditis		–	16

*Major and minor criteria that applied to patients with positive and negative ^99m^Tc-Besilesomab-SPECT/CT and their classification according to the modified Duke Criteria are shown*.

**Figure 1:**
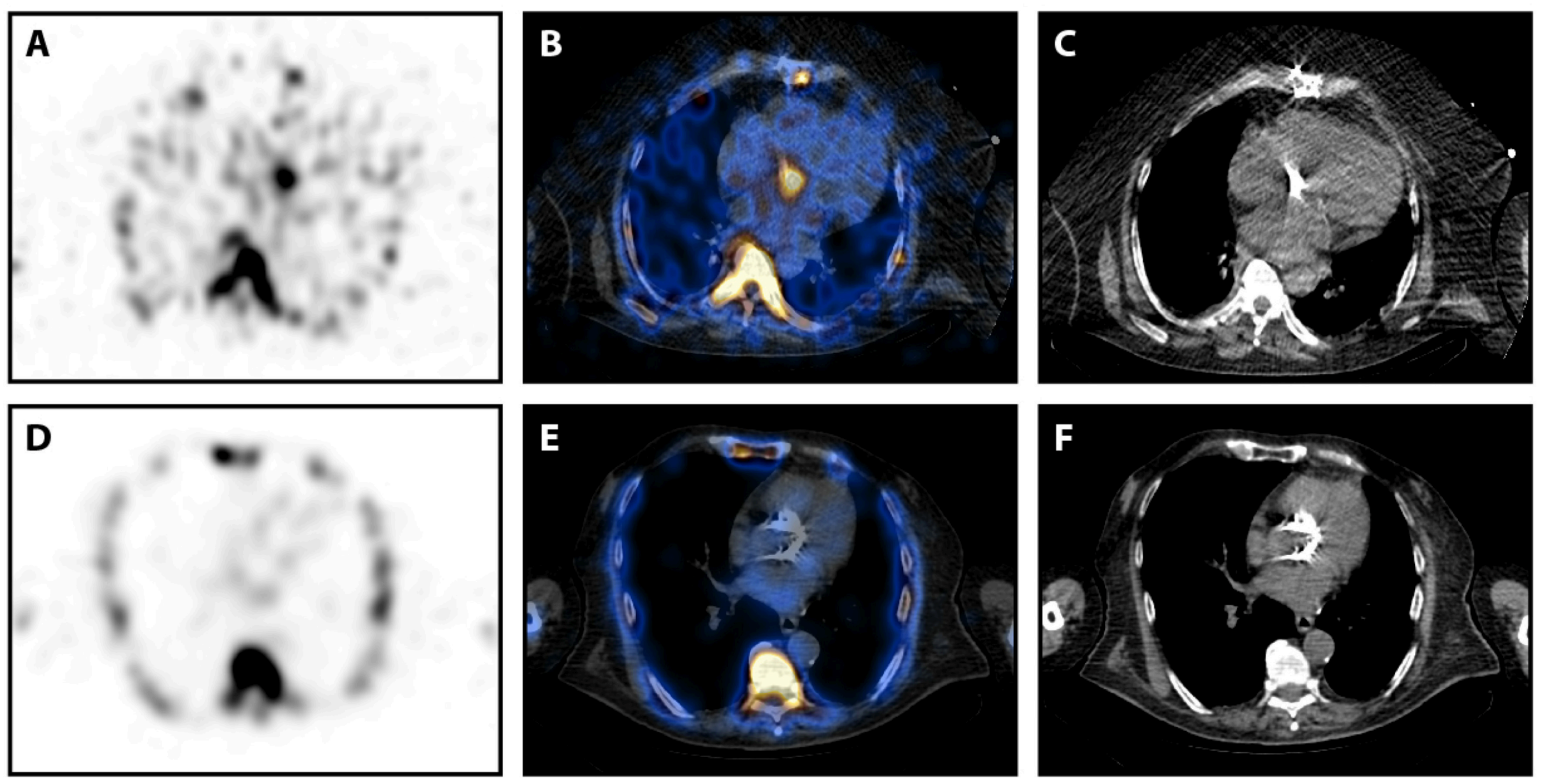
^99m^Tc-Besilesomab-SPECT/CT in patients with aortic valve prostheses. **(A–C)** SPECT/CT images in transversal view of a 63-years old male patient with suspected prosthetic valve infection 5 years after an aortic valve replacement. Blood cultures detected *Staphylococcus aureus* and TEE suspected an abscess formation. SPECT showed a distinct focal uptake at the aortic valve level **(A)**. This focal uptake could be localized around the prosthetic valve in fused images **(B)**. **(C)** Non-fused CT image of the prosthetic valve. Pathology results confirmed infection of the prosthesis. **(D,E)** SPECT/CT images in transversal view of an 82-years old male patient with suspected prosthetic valve infection 3 years after a transcatheter aortic valve implantation. Blood cultures were negative and TEE was inconclusive. SPECT did not detect any pathological uptake **(D)**, especially around the aortic valve replacement **(E)**. **(F)** Non-fused CT image of the prosthetic valve. Endocarditis was excluded by clinical findings according to the Duke criteria.

^99m^Tc-Besilesomab-SPECT/CT was positive in 7 cases showing focal uptake around aortic valve replacements (*n* = 5; Figure 1), at a native aortic valve and an infected thrombus in the right atrium/around the tricuspid valve. TBR was 3.27–12.96 (mean 5.77). All cases were true positive. *N* = 2 patients were confirmed as true positive by pathological findings. Blood cultures detected *Staphylococcus aureus* (*n* = 2), *Staphylococcus epidermidis*, and *Enterococcus faecalis*. Blood cultures of 3 cases were negative. TEE was positive in 5/7 cases showing vegetation, abscesses (*n* = 2) and paravalvular thickening (*n* = 2). Two cases were negative in TEE. CT showed a paravalvular lesion in one case. According to the modified Duke Criteria diagnosis of endocarditis was “definitive” in 5 patients and “possible” in 2 patients (Table 2). Both cases with a “possible” endocarditis diagnosis showed distinct focal ^99m^Tc-Besilesomab uptake around an aortic valve replacement with a TBR of 6.26 and 5.45. Due to the SPECT/CT results, diagnosis was changed to “definitive” in both cases. Treatment of the patients included surgery (*n* = 2) and antibiotics (*n* = 5).

Remaining 19/26 patients did not show any pathological uptake in ^99m^Tc-Besilesomab-SPECT/CT. TBR was 1.03–1.71 (mean 1.45). All 19 cases were true negative for endocarditis. Histology confirmed findings as true negative in *n* = 4 cases. TEE was available in 18/19 cases and showed negative findings in 9/18 cases. In 3/18 cases TEE showed vegetations (*n* = 3) or abscess formation. Six cases showed inconclusive findings in TEE. Blood cultures were available in 17/19 cases and were positive in 5 patients (*Staphylococcus aureus* (*n* = 3), *Enterococcus faecalis* (*n* = 2). These patients were diagnosed with spleen and subphrenic abscess, soft tissue infection, occult sepsis (“rejected endocarditis” according to modified Duke Criteria'), urinary tract infection, and pulmonary infection. Therefore, positive blood cultures were caused by other conditions than endocarditis. Blood cultures of remaining 12 patients were negative. One patient underwent ^18^F-FDG-PET/CT which was negative. According to the modified Duke Criteria diagnosis of endocarditis was “possible” in 3 patients and “rejected” in 16 patients (Table 2). Cases with a “possible” endocarditis diagnosis did not show any pathological intracardial uptake in ^99m^Tc-Besilesomab-SPECT/CT with a TBR < 1.59. Due to the SPECT/CT results, diagnosis was changed to “rejected endocarditis” in both cases.

**Figure 2:**
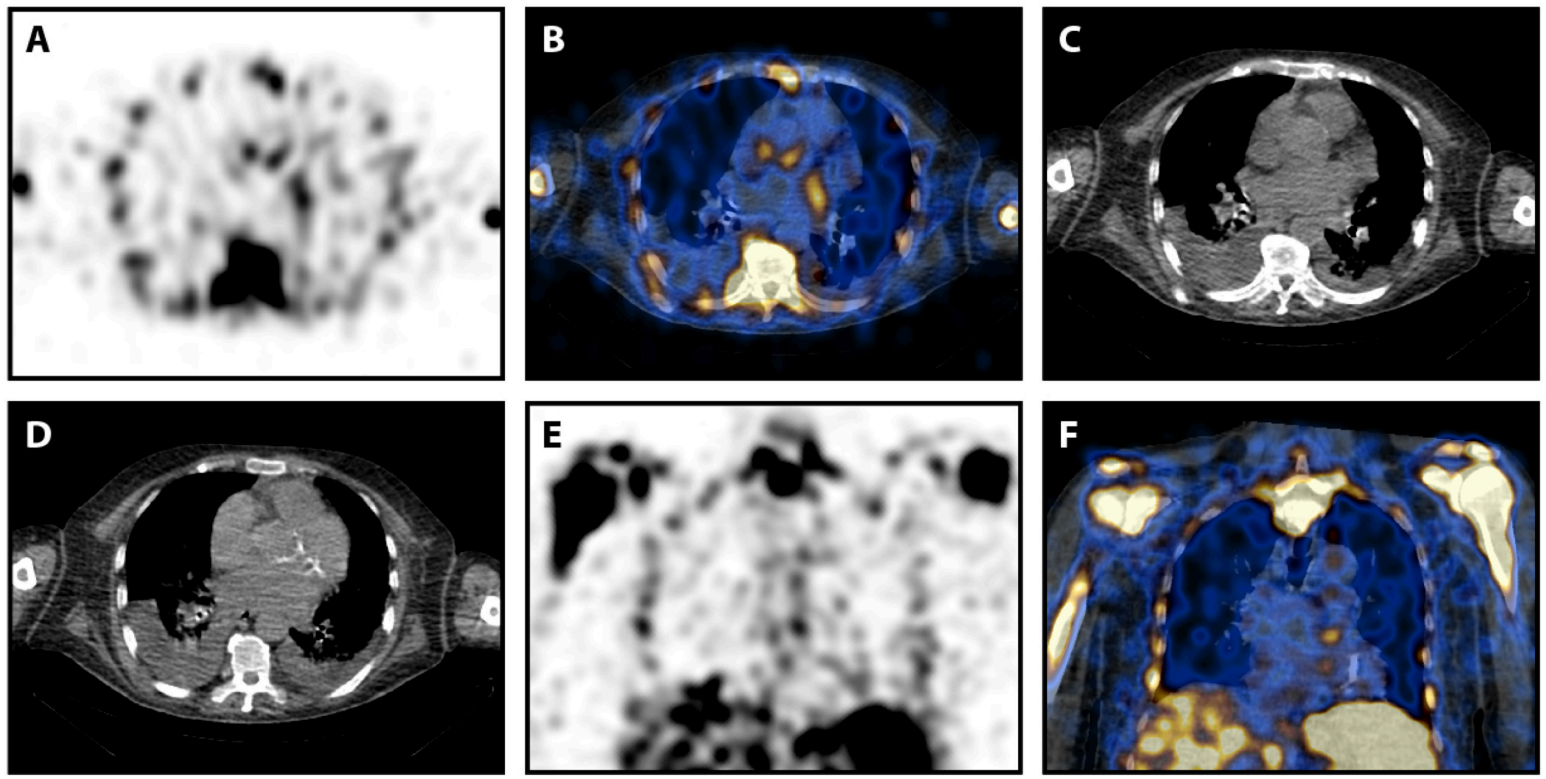
^99m^Tc-Besilesomab-SPECT/CT in a patient with suspected prosthetic valve infection and intra-observer difference. Images of a 78-years old male patient with suspected prosthetic valve infection. Blood cultures detected *Enterococcus faecalis* and TEE was negative. SPECT showed a trifocal uptake at the aortic valve level **(A)**. This focal uptake was localized slightly cranial of the prosthetic valve in fused images **(B)**. **(C)** Non-fused CT image at the same level as **(B)**. **(D)** Non-fused CT image of the prosthetic valve caudal of **(D)**. Focal uptake was also visible in coronal view **(E,F)**. Observer 1 scored the focal uptake “2” while observer 2 scored it “1.” The patient had a fever above 38°C for 4 weeks. Clinical inspection detected Osler's nodes on the hands. Endocarditis was clinically confirmed according to Duke criteria.

A discrepancy between the observers occurred in one patient with a myelodysplastic syndrome and an aortic valve prosthesis. SPECT/CT was visually scored “2” by observer 1 and “1” by observer 2 (Table 2). ^99m^Tc-Besilesomab-SPECT/CT showed a focal uptake around the aortic valve replacement with a TBR of 5.72 (Figure 2). TEE was negative. *Enterococcus faecalis* was detected in blood cultures. The patient had a fever above 38°C for 4 weeks. Clinical inspection detected Osler's nodes on the hands. Diagnosis of endocarditis was “definite” according to the modified Duke Criteria.

**Figure 3:**
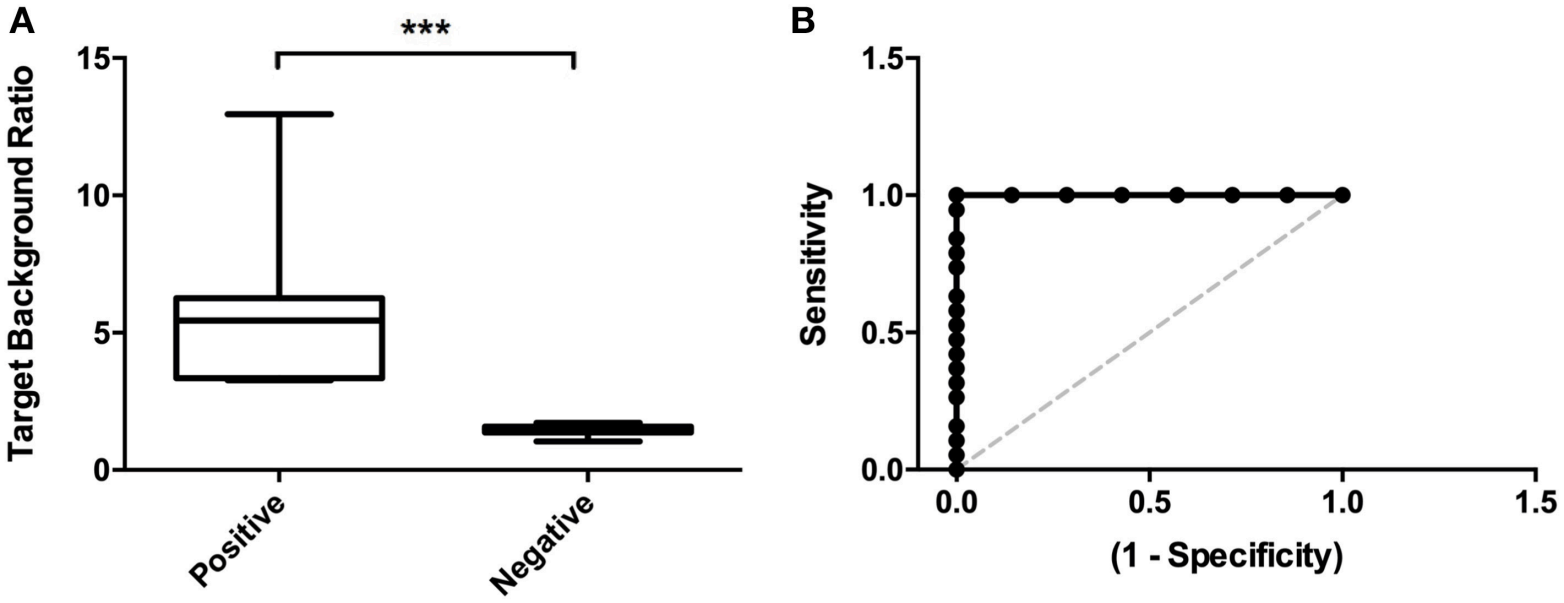
Image analyses. **(A)** Target Background Ratios were significantly higher in positive scans compared to scans that were visually rated negative. **(B)** ROC curve analysis detected an optimal cut-off value for TBRs < 0.67 with 94.74% sensitivity and 100% specificity. ^***^p < 0.001.

TBRs were significantly higher in patients that were visually scored positive (3.27–12.96; mean 5.77) compared to negative cases (1.03–1.71; mean 1.45; *p* < 0.0001; Figure 3A). ROC curve analysis showed the optimal cut-off point for the TBR < 1.69 with 94.74% sensitivity and 100% specificity and AUC of 1 (Figure 3B). Visual scores differed between observers in 4 cases that were ruled negative and in one case (as described above; Table 3) that was positive. Visual scores correlated with Target Background Ratios (*r* = 0.66;).

**Table 3:** ^99m^Tc-Besilesomab-SPECT/CT results.

	**Observer 1**	**Observer 2**
Patients	26	26
Positive scans	7	6
TP	7	6
FP	0	0
Negative scans	19	20
TN	19	19
FN	0	1

*Two different blinded observers reviewed images separately. TP, true positive; FP, false positive; TN, true negative; FN, false negative*.

Sensitivity of ^99m^Tc-Besilesomab-SPECT/CT was 86–100% and specificity was 100%. Inter-observer agreement of the scores of all scans (0–3) was good (weighted kappa = 0.76) and very good of deciding whether a scan was positive or negative (kappa = 0.9).

## Discussion

Reliable diagnosis of infectious endocarditis can be challenging. Infective endocarditis still displays a life-threatening disease with high mortality rates. Clinical diagnosis of endocarditis relies on the Duke Criteria considering the detection of a pathogen in blood cultures and positive echocardiography as major criteria and unspecific symptoms of infection, predisposing heart conditions or vascular and immunologic phenomena as minor criteria. The “modified Duke Criteria” as recommended by the American Heart Association and the European Society of Cardiology define a “definite” endocarditis diagnosis if histology or cultures of an intra-cardiac lesions are positive or different combinations of major and minor criteria are present (1, 3, 4).

TEE is an important part of the Duke criteria. It can detect vegetations, destructive valve lesions or abscess formation in order to support the diagnosis of endocarditis (8). However, about 15% of infectious endocarditis cases are false negative in TEE (9). In our cohort TEE was false negative in 29% of patients with confirmed endocarditis and false positive or inconclusive in 50% of patients with excluded endocarditis. These results might be explained by the high rate of patients with prosthetic valves in our cohort. In our study 50% of cases were in a post-interventional state. TEE results can be impaired by artifacts caused by prosthetic heart valves or intracardiac devices reducing sensitivity and specificity of the method. Infectious endocarditis which is associated with valve prosthesis represents a frequent and often severe form of endocarditis accounting for around 10–30% of cases. In these cases, TEE shows even a lower sensitivity compared to native valve infections (8). Furthermore, calcifications or thickened valves can be misinterpreted as vegetations leading to false-positive results in echocardiography (10). Overall, echocardiography displays an essential tool of the clinical diagnosis of infectious endocarditis but cannot alone definitively confirm or exclude suspected endocarditis in all situations.

In the 2015 guidelines of the European Society of Cardiology for the management of infective endocarditis further cardiac imaging methods are proposed in challenging cases with “possible” diagnosis of infectious endocarditis according to the modified Duke Criteria. In cases of suspected prosthetic valve infections radionuclide imaging with ^18^F-FDG-PET or SPECT with radiolabeled leucocytes were suggested.

Granulocyte imaging with the anti-CD66/67 antibody ^99m^Tc-Besilesomab displays another techniques that can help in those cases. So far, only a small amount of studies on this topic, carried out in the 1990s, exists and the use of ^99m^Tc-Besilesomab scintigraphy was limited to certain centers, including our institution, even though the tracer is routinely available.

The first study was published by Munz et al. (11). Using SPECT imaging 24 h post injection sensitivity of 79% and specificity of 82% was detected in a small cohort of 20 patients. The largest number of patients has been studied so far by Morguet et al. (12, 13). A total of 110 patients with suspected endocarditis were examined with ^99m^Tc-Besilesomab-SPECT 20 h post injection. Sensitivity was 78–79% and specificity was 82–85%. A combination of TEE and ^99m^Tc-Besilesomab-SPECT showed a sensitivity of 100%. In 16 cases a follow up of ^99m^Tc-Besilesomab-SPECT was performed and results were concordant to the clinical follow up (12, 13). Unpublished retrospective data of our facility using ^99m^Tc-Besilesomab-SPECT are in line with previous results with sensitivity of 89% and specificity of 100% (Bouter et al. unpublished).

Becker et al. examined patients with fever of unknown origin including an endocarditis subgroup with ^99m^Tc-Besilesomab. However, only planar imaging was used. In this study only 2 of 9 patients with endocarditis were true positive (14). Due to relatively high uptake in the red bone marrow SPECT is mandatory in order to assess the heart and detect endocarditis.

Other nuclear medicine methods were used in infectious endocarditis so far as well. Earlier studies could not show a benefit of ^111^In-labeled leucocyte scintigraphy as sensitivity was only 46% (15). Two studies with ^99m^Tc-HMPAO-labeled leucocytes using SPECT/CT in patients with suspected infectious endocarditis and suspected prosthetic valve endocarditis are published so far (16, 17). Erba et al. showed a sensitivity of 90% and specificity of 100% in a cohort of 131 patients with suspected endocarditis excluding patients with cardiac devices (16). Hyafil et al. examined 42 patients with prosthetic valve endocarditis with inconclusive findings in TEE and leucocyte scintigraphy changed the patient management in 29% of cases (17).

To our best knowledge, no studies on SPECT/CT imaging with ^99m^Tc-Besilesomab are published so far. Our results build on earlier ^99m^Tc-Besilesomab-SPECT results. However, ^99m^Tc-Besilesomab-SPECT/CT showed a better diagnostic accuracy (sensitivity and specificity up to 100%). ^99m^Tc-Besilesomab-SPECT/CT changed the clinical diagnosis of “possible” endocarditis to “definite” endocarditis in two cases and from “possible” to “rejected” in three cases. In our study (with a limited number of patients) semi-quantitative evaluation showed that a target-to-background-ratio of 1.69 might be a useful cut-off point in order to exclude florid endocarditis. This could be a starting point for future prospective studies.

However, compared to ^99m^Tc-Besilesomab-SPECT alone, the major advantage of the hybrid imaging method seems to be the added anatomical information from CT images. SPECT/CT can identify focal uptake and correctly pinpoint the site of the uptake in CT. Therefore, it helps discriminating between involvement of a cardiac valve, device cable, or infected thrombus by correctly assessing the site of infection. Furthermore, native and artificial heart valves can be localized in CT images and surrounding areas can be scanned for elevated uptake in fused images. The added anatomical information might lead to an improved diagnostic accuracy. Reliable anatomical localization can also help in surgery planning.

Our results are in line with earlier studies using ^99m^Tc-HMPAO-labeled leucocyte SPECT/CT. A clear advantage of ^99m^Tc-Besilesomab in comparison to *ex vivo* labeling of leucocytes is the timesaving approach which avoids handling of blood products.

One observer was false negative in one case in our study. This case was true positive in the evaluation of the second observer (Figure 2). Misestimation of one observer might be explained by a slightly lower TBR compared to other cases. The patient had a myelodysplastic syndrome which might be accountable for the lower uptake due to increased bone marrow uptake and a lower number of functional granulocytes (18, 19). The observed focal uptake did not perfectly match the prosthetic valve in CT. However, interfering factors in myocardial imaging as cardiac (and respiratory) motion have to be taken into account as a possible limitation of the method. ECG-gated imaging might be useful in order to resolve these issues in future studies.

All other negative cases were true negative. However, sensitivity of granulocyte imaging can be affected in certain situations that impair immune response including microorganisms that escape immune cells by extracellular proteases or biofilm formation (as *Enterococcu*s and *Candida* infections) as well as antibiotic treatment leading to lower TBR (20, 21).

Furthermore, spatial resolution of SPECT imaging is limited even with the use of advanced SPECT/CT scanners. PET/CT, which was also included in the 2015 guidelines of European Society of Cardiology for the diagnosis of infective endocarditis, might be used to overcome this issue. A meta-analysis of 2018 detected a pooled sensitivity of ^18^F-FDG-PET/CT for the diagnosis of infectious endocarditis of 81% and a pooled specificity of 85% (22). However, several limitations of ^18^F-FDG-PET/CT have to be considered. Specificity of ^18^F-FDG uptake can be limited due to several factors as false positive uptake in papillary muscles, reconstruction artifacts of myocardial uptake or increased post-interventional unspecific uptake. Especially in the cardiac region ^18^F-FDG-PET/CT can be aggravated by different metabolic conditions making a correct preparation of the patients (fasting, specific diet, glucose or insulin clamp) essential. Also, the limited availability in smaller clinics and high costs of PET scanners has to be taken into account.

Both ^18^F-FDG-PET/CT and leucocyte SPECT, are useful modalities in the diagnostic workup of infectious endocarditis. Both methods, with its strengths and limitations, should be considered in uncertain possible endocarditis cases. Future studies comparing ^18^F-FDG-PET/CT and leucocyte SPECT/CT would be of great interest. Specific PET/CT imaging with positron emitting labeled leucocytes might be a further solution in the future. Dumarey et al. showed significant uptake in the aortic valve of a patient with infectious endocarditis with ^18^F-FDG-labeled leucocytes (23). However, the usage of ^18^F-tracers is limited by its short physical half-life, which rules out later imaging in order to assess kinetics of leucocyte migration.

Limitations of our study include the retrospective setting and the relatively small patient number. Further studies have to be carried out in order to build on the current findings.

## Conclusion

^99m^Tc-Besilesomab-SPECT/CT is a useful imaging method in order to confirm or exclude suspected endocarditis, especially in difficult cases with prosthetic valves or cardiac devices with inconclusive findings in echocardiography. It should be included to the guidelines for the management of infective endocarditis as an addition to conventional diagnostics using the modified Duke Criteria in order to reduce the rate of false diagnoses. Hybrid imaging with SPECT/CT helps to exactly localize focal uptake to a certain valve, prosthesis, or device cable and is superior to SPECT imaging.

## Author Contributions

CB analyzed data, designed the project, and wrote the manuscript. CS and BM participated in the discussion of the results. JM conceived the project and contributed to discussion of the results. All authors contributed to revising the manuscript and approved the final version.

### Conflict of Interest Statement

The authors declare that the research was conducted in the absence of any commercial or financial relationships that could be construed as a potential conflict of interest.
